# Heat of Formation of Decaborane

**DOI:** 10.6028/jres.064A.052

**Published:** 1960-12-01

**Authors:** Walter H. Johnson, Marthada V. Kilday, Edward J. Prosen

## Abstract

The heat of formation of crystalline decaborane has been determined by calorimetric measurement of the heat of decomposition:

$$\begin{array}{c} B_{10}H_{14}\left(c\right)\to 10B\left(\text{am}\right)+7H_{2}\left(g\right),\\ \Delta H\left(25\;{}^{\circ}C\right)=82.8\pm 5.9\;\text{kj}/\text{mole},\;\left(19.8\pm 1.4\;\text{kcal}/\text{mole}\right). \end{array}$$

With the heat of transition of crystalline to amorphous boron taken as 0.40 kcal/mole, we obtain for the heat of formation of decaborane:

$$\Delta {\text{Hf}}_{298.15}^{{}^{\circ}}=-66.1\;\text{kj}/\text{mole},\;\left(-15.8\;\text{kcal}/\text{mole}\right).$$

## 1. Introduction

Combustion methods afford a convenient and precise method for determination of the heats of formation of a large number of compounds. In the case of decaborane, however, preliminary measurements of the heat of combustion of decaborane have been found to be very unreliable. Combustion experiments in an oxygen bomb failed to yield results of the required precision; the sample detonated in practically all cases and the combustion products included varying amounts of elemental boron and higher boron hydrides. There was also some uncertainty in the amount of dissolved boric acid and the possibility of the presence of boron oxides. In general, the experiments were performed with the decaborane sample in the form of a pellet. In some cases, in which the sample was burned in the form of loose crystals or of crystals intermixed with quartz fibers, the combustion residue also included decaborane. In other experiments a solution of decaborane in toluene was burned; the resulting quantity of carbon dioxide, however, indicated the formation either of elemental carbon or of boron carbide.

The quantity of boric acid found in the water-soluble portion of the combustion products varied from 75 to 95 percent of the theoretical amount calculated from the mass of sample. The resulting heat of combustion of decaborane (to crystalline boric oxide and liquid water) was −2004 kcal/mole. A reasonable estimate of the probable error in the heat of combustion of decaborane, as determined by the oxygen bomb process, is about 1 percent. In view of this information, it is obvious that the heat of formation calculated from the heat of combustion is subject to a relatively large uncertainty.

Liebhafsky [1]^1^ also determined the heat of combustion of decaborane. The heat of formation of decaborane [2] was taken as 8 kcal/mole, calculated from his results.

The decomposition of crystalline decaborane into amorphous boron and gaseous hydrogen proved to be the best method for calorimetric determination of the heat of formation. The results of this investigation are presented here.

## 2. Source and Purity of Materials

The decaborane was obtained from the General Electric Research Laboratory through the courtesy of A. E. Newkirk and A. L. Marshall. It was prepared by the pyrolysis of diborane and purified by several vacuum sublimations, the last two of which produced materials with no measurable difference in melting point. Prior to this investigation it was further purified by two successive vacuum sublimations and stored in a desiccator over magnesium perchlorate.

A mass spectrometric analysis of the helium^2^ used showed it to contain less than 0.01 volume percent of air; the helium was further treated as described in the next section of this paper.

## 3. Apparatus

The isothermal jacket calorimeter has been described previously [3]. The calorimetric system consisted of the nickel-plated copper calorimeter can and cover into which was placed a weighed quantity of water, the calorimetric reaction vessel, and the platinum resistance thermometer. The apparatus for measuring calorimeter temperatures and electrical energy have been described previously [3, 4, 5].

**Figure 1:**
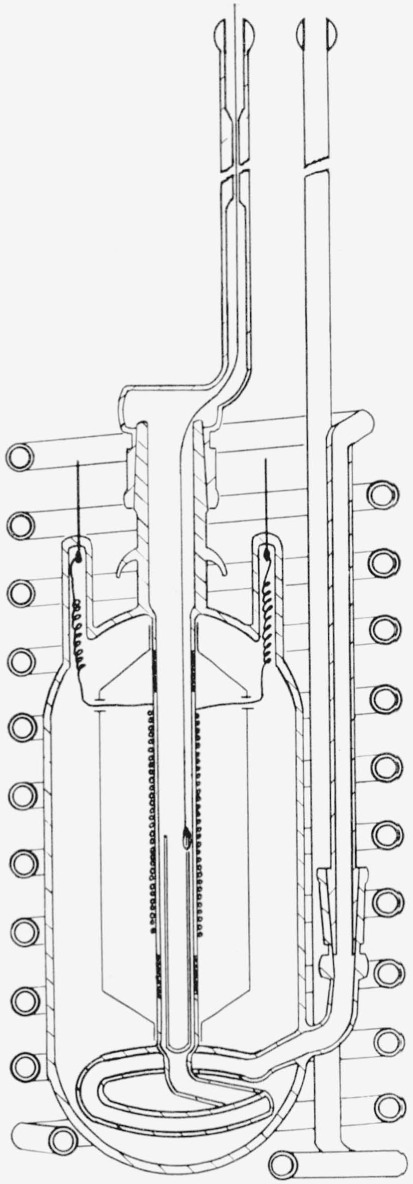
Decomposition vessel.

A diagram of the calorimetric decomposition vessel is given in figure 1. The vessel consisted of a quartz tube, wound with a 183-ohm nichrome heating coil, surrounded with a silver radiation shield, and a vacuum jacket. The decaborane sample was placed in the Vycor crucible at the lower end of the quartz tube. Helium entered at the top through a coarse capillary immediately above the vessel, flowed around the crucible and out through the glass helix. A nichrome wire attached to the crucible was led out of the vessel through the inlet tube and fastened to a circularly ground stopcock in the helium line. By turning the stopcock, the crucible could be raised without interrupting the flow of helium or permitting air to enter the vessel. An aluminum cover (not shown in the diagram) which fitted over the top of the vessel served to prevent loss of energy by radiation through the opening in the calorimeter can cover.

**Figure 2:**
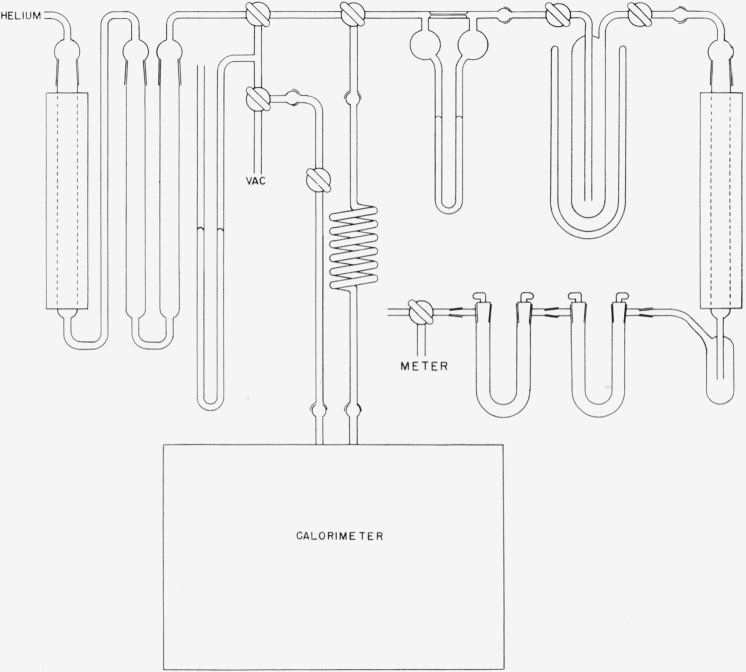
Gas train used in decomposition of decaborane.

A diagram of the gas train is shown in figure 2. The helium was purified by passing it successively over titanium, copper, and copper oxide in a Vycor tube which was heated to 600 °C and through absorbers containing Ascarite, anhydrous magnesium perchlorate, and phosphorus pentoxide. The exit gases were passed successively through a capillary flowmeter, a trap cooled with liquid nitrogen, a Vycor tube containing copper oxide at 600 °C, two water-absorption tubes, each containing magnesium perchlorate and phosphorus pentoxide, and a recording gas-volume meter.

The capillary flowmeter served for the approximate adjustment of the rate of flow of helium and also to indicate the rate of the decomposition process. The cold trap was included to collect any traces of volatile boron hydrides. The hydrogen evolved in the decomposition was burned to water in the copper-oxide heater and carried, as vapor, into the weighed absorption tube by the stream of dry helium. The portion of the water which collected as liquid in the bulb below the heater was evaporated by warming the bulb slightly. The second absorption tube served only to protect the first from moisture in the atmosphere. The total quantity of helium was measured by means of the recording gas-volume meter at the exit end of the gas train. The approximate temperature of the helium which entered the system during the experiment was measured by means of a thermometer placed in contact with the inlet tube of the decomposition vessel.

## 4. Procedure

The decaborane sample was placed in the weighed Vycor crucible and melted by heating with care in a helium atmosphere. When cool, the crucible plus sample was weighed, transferred to the decomposition vessel, and the vessel assembled. The vessel was placed in the calorimeter can, which contained 5,653 g water at 25.0 °C. The calorimeter assembly was lowered into the water bath, the temperature of which was adjusted to 27.0 °C and maintained constant to ±0.001 °C.

Air was removed from the vessel by evacuation and by flushing with helium. Thermal equilibrium was established after about 20 minutes; the helium was then directed to by-pass the decomposition vessel and temperatures were observed at 2-minute intervals during a 20-minute initial rating period. Helium was then directed through the vessel and recording gas-volume meter, and an electric current was passed through the heating coil. About 6 minutes were required for the quartz tube to attain the decomposition temperature of about 650 °C. The crucible was then raised very slowly by turning the circularly ground stopcock until hydrogen began to be evolved as indicated by the increase in the reading on the capillary flowmeter. When the evolution subsided the crucible was again raised slightly; this process was continued until the crucible was located completely within the heated zone of the decomposition vessel. A period of about 6 minutes was required for elevation of the crucible and an additional period of 6 to 8 minutes was allowed for completion of the decomposition process. The heating current was then interrupted and the flow of helium through the vessel was continued for an additional 40 minutes to re-establish thermal equilibrium.

Measurements of the current through the heating coil and the potential drop across the coil were made alternately at 1-minute intervals during the heating period. Calorimeter temperatures were observed at 1-minute intervals during the entire 1-hour “reaction” period. The helium flow was then directed to by-pass the decomposition vessel and the recording gas-volume meter, and calorimeter temperatures were observed at 2-minute intervals during a final 20-minute rating period.

After the experiment the decomposition vessel was removed from the calorimeter and disassembled. Although most of the boron remained in the crucible, a significant amount was transported to the walls of the vessel. It was necessary to place a plug of glass wool in the helix in order to prevent some of the boron from being carried out of the calorimeter by the helium and hydrogen. Some small crystals of decaborane, apparently formed by sublimation, were found in the upper part of the vessel. These usually became dislodged and fell into the crucible during removal of the crucible from the vessel. It was therefore not possible to collect, quantitatively, all of the boron produced in the decomposition.

The quantity of decaborane decomposed was calculated from the weight of hydrogen, as water, produced by the process. The accuracy of this determination was checked by dissolving a weighed quantity of zinc in dilute sulfuric acid while a slow stream of helium was bubbled through the solution and directed through the gas train. The water vaporized from the solution was retained in the cold trap; the mass of water produced by burning the hydrogen agreed within 0.2 percent with the theoretical quantity calculated from the mass of zinc.

There was usually a small quantity of nonvolatile brownish material remaining at the top of the crucible which was assumed to consist of higher boron hydrides. The analysis of this material was complicated by contamination with decaborane as described above. An attempt was made to remove the decaborane by placing the residue in a tantalum boat and heating slowly to 300 °C in a stream of helium. The material which remained was weighed and then heated to 800 °C for 1 hour in vacuum. The loss in weight resulting from this treatment may be assumed to be due to removal of hydrogen from the residue. The amount of hydrogen remaining in the residue was indicated by this procedure to be approximately 0.2 percent by weight which corresponds to slightly less than 2 percent of the total hydrogen in the original material.

The energy equivalent of the calorimeter was determined in a separate series of experiments which were performed in the same manner as the decomposition experiments. In each case the calorimeter system was made to be nearly identical with that used in the decomposition experiments except that the decaborane sample was not present.

## 5. Results and Calculations

The molecular weights of boron, hydrogen, and water have been taken as 10.82, 2.016, and 18.016 g/mole, respectively. The following mean heat capacities in j/° C mole for the range of 25 to 27 °C were used]:


He(gas)20.8[2]H_2_(gas)28.8[2]B_10_H_14_(c)219.7[6]B(amorph)11.1[7].

The results of the electrical calibration experiments are given in table 1, where Δ*Rc* is the corrected temperature rise [4, 8, 9], *E* is the quantity of electrical energy introduced, *q*_He_ is a correction applied to convert the temperatures of the inlet and exit helium to 25 °C, and *E_s_* is the heat capacity of the standard calorimetric system obtained by the relationship:

$$\frac{E+q_{H_{e}}}{\Delta Rc}=E_{s}.$$

The results of the decaborane decomposition experiments are given in table 2. The quantities $q_{H_{e}}$, $q_{H_{2}}$, $q_{B}$, and $q_{B_{10}H_{14}}$ are the corrections required to convert the actual decomposition process to the isothermal process at 25 °C. The change in enthalpy corresponding to the decomposition process was determined for each experiment by the relationship:

$$\Delta H\left(25\;{}^{\circ}C\right)=\frac{E+q_{H_{e}}+q_{H_{2}}+q_{B}+q_{B_{10}H_{14}}-\Delta Rc\left(E_{s}\right)}{\text{mole}\;B_{10}H_{14}}.$$

For conversion to the conventional thermochemical calorie, 1 calorie has been taken as equivalent to 4.1840 joules. The mean of the values given for ΔH in table 2 corresponds to the process:

$$\begin{array}{l} B_{10}H_{14}\left(c\right)\to 10B\left(\text{amorph}\right)+7H_{2}\left(g\right)\\ \Delta H\left(25\;{}^{\circ}C\right)=82.8\pm 5.9\;\text{kj}/\text{mole},\\ \;=19.8\pm 1.4\;\text{kcal}/\text{mole}. \end{array}$$

With the value 0.40 kcal/mole taken for the heat of transition of crystalline to amorphous boron [2], there is obtained for the heat of formation of decaborane:

$$\begin{array}{l} \Delta \text{Hf}\;{}^{\circ}\left(25\;{}^{\circ}C\right)=-66.1\;\text{kj}/\text{mole},\\ \;=-15.8\;\text{kcal}/\text{mole}. \end{array}$$

The heat of the transition from amorphous boron to the stable crystalline form has not been determined. Some preliminary measurements in this laboratory have indicated that it is small. The enthalpy change for the process:

B(c)→B(amorph),

has been estimated to be 0.40 kcal/mole [1]. No uncertainty has been assigned to this arbitrary value.

There is also some ambiguity in regard to the thermodynamic state of the amorphous boron obtained from the decomposition of the hydride. Several types of amorphous boron may exist. In addition, the effect of the particle size is not known. The decomposition of the hydride, however, is believed to be a step-wise process and the nature of the resulting amorphous product, obtained at a given temperature, is expected to be independent of the boron-hydrogen ratio of the initial hydride.

The heat of formation of decaborane may be combined with the heats of formation of boric oxide [3] and water [2] to obtain the heat of combustion:

$$\begin{array}{c} B_{10}H_{14}\left(c\right)+11O_{2}\left(g\right)\to 5B_{2}O_{3}\left(c\right)+7H_{2}O\left(\text{liq}\right),\\ \Delta \text{Hc}{}^{\circ}\left(25\;{}^{\circ}C\right)=-1989.1\pm 4.0\;\text{kcal}/\text{mole}. \end{array}$$

The uncertainties assigned to the values given in this paper have been taken as twice the standard deviation of the means of the calibration and decomposition experiments, combined with reasonable estimates of all other known sources of error.

## 6. Discussion

The heats of formation of diborane and pentaborane were determined in a similar manner by measurement of the heats of decomposition of the vapors [10]. In each of these cases the decomposition process was accompanied by the evolution of energy; in the case of decaborane, however, the process involves the absorption of energy. In every case the quantity of material decomposed was determined by measurement of the amount of hydrogen evolved. This method has the advantage of limiting the error introduced by incomplete decomposition to the difference between the mean energies of the broken and unbroken boron-hydrogen bonds multiplied by the fraction of unbroken bonds.

There is a possibility that some of the hydrogen may remain adsorbed on the amorphous boron at the decomposition temperature, but this quantity is believed to be small. Samples of amorphous boron produced by pyrolysis of diborane at 600 °C were heated to 850 °C in vacuum with no significant loss of weight.

Simons, Balis, and Liebhafsky [11] made an elemental analysis by heating decaborane in sealed palladium tubes to 900 °C in vacuum and were able to remove 99.99 percent of the available hydrogen. In view of these data it appears that any error introduced by incomplete decomposition or by adsorption of hydrogen on the amorphous boron residue is small in comparison with the uncertainty assigned to the experimental values.

## Figures and Tables

**Table 1:** Results of the electrical calibration experiments

Experiment	Δ*Rc*	*E*	*q*_He_	*Es*

	*ohm*	*j*	*j*	*j*/*ohm*
1	0.183984	37350.3	1.0	203014
2	.161826	32848.2	2.8	203002
3	.169807	34488.3	2.0	203115
4	.188565	38301.9	2.6	203137
5	.235845	47888.1	3.5	203064
6	.256337	52016.6	3.1	202923

Mean				203042
Standard deviation of the mean				±32.4

**Table 2:** Results of the B_10_H_14_ decomposition experiments

Experiment	Δ*Rc*	*E*	*q*_He_	*q*h_2_	*q*b	*q*b_10_h_14_	Moles B_10_H_14_	ΔH (25 °C)

	*Ohm*	*j*	*j*	*j*	*j*	*j*		*kj*/*mole*
1	0.178111	36330.0	2.9	−0.1	−0.4	−0.5	0.0018321	91.64
2	.180650	36806.8	1.8	.0	−.4	−.5	.0016701	76.76
3	.178745	36422.6	2.2	.0	−.3	−.5	.0016462	79.76
4	.176884	36023.9	2.2	−.1	−.3	−.4	.0016111	68.52
5	.215651	43948.5	3.6	−.2	−.4	−.4	.0017796	92.66
6	.236409	48154.4	2.8	−.3	−.3	−.4	.0016620	93.38
7	.213561	43512.3	1.6	−.2	−.4	−.5	.0017852	84.53
8	.238218	48496.6	2.7	−.3	−.4	−.4	.0017660	73.56
9	.221232 a	45046.1	3.5	−.2	−.3	−.4	.0016111	84.79

Mean								82.84
Standard Deviation of the mean								±2.96

a There is a correction of −33.0 j/ohm in the energy equivalent of the calorimeter system used for this experiment.

## Footnotes

1 Figures in brackets indicate the literature references at the end of this paper.

2 This analysis was performed by V. H. Dibeler of the Mass Spectrometry Section of the Atomic and Radiation Physics Division.
